# Clinicopathological Analysis and Surgical Outcome of Eyelid Malignancies: A Study of 332 Cases

**DOI:** 10.1155/2022/4075668

**Published:** 2022-02-18

**Authors:** Syeed Mehbub Ul Kadir, Mukti Rani Mitra, Riffat Rashid, Murtuza Nuruddin, Md. Kamrul Hassan Khan, Golam Haider, Mst. Sayedatun Nessa

**Affiliations:** ^1^Training and Academic, Sheikh Fazilatunnesa Mujib Eye Hospital and Training Institute, Gopalgonj, Bangladesh; ^2^Department of Ophthalmology, Dhaka Medical College Hospital, Dhaka, Bangladesh; ^3^Department of Ocular Oncology and Oculoplasty, Ispahani Islamia Eye Hospital and Institute (IIEHI), Dhaka, Bangladesh; ^4^Department of Oculoplasty, Chittagong Eye Infirmary and Training Complex (CEITC), Chittagon, Bangladesh; ^5^Head of the Department of Ophthalmology, Combined Military Hospital, Dhaka, Bangladesh; ^6^Medical Education, Bangladesh Eye Hospital and Institute, Dhaka, Bangladesh; ^7^Department of Pathology, Northern International Medical College, Dhaka, Bangladesh

## Abstract

**Background:**

Eyelid tumours are common in our ophthalmic practice. Malignancy cases account only for one-fourth of all eyelid tumours. The most aggressive eyelid malignancy is sebaceous gland carcinoma, but its occurrences are rare in western countries. We found sebaceous gland carcinoma is as common as basal cell carcinoma in our clinical practices. Hence, it is essential to build awareness about the more aggressive eyelid malignancies to reduce morbidity and mortality.

**Aim:**

To assess the relative frequency of eyelid malignancies in the Bangladesh population, state their clinical features and outcome of management strategies and build awareness about the more aggressive eyelid malignancies to reduce morbidity and mortality.

**Methods:**

This was a retrospective case series study of 332 patients in Bangladesh. This study analyzed all the recorded data of the histologically proven primary eyelid malignancies and followed them up for at least six months from 2014 to 2019 (6 years). All patients were managed by surgical excision with tumor-free margins verified on histopathology, either the frozen section or excision biopsy with 2–3 mm microscopic view of normal tissue followed by eyelid reconstruction. Computer-based statistical software SPSS was used for the analysis, and an appropriate test of significance (chi-square) was used for the statistical analysis.

**Results:**

Sebaceous gland carcinoma (SGC) was the highest in occurrence, at 42%, followed by 38% basal cell carcinoma (BCC), 18% squamous cell carcinoma (SqCC), and 02% malignant melanoma (MM). The mean age at presentation of SGC, BCC, SqCC, and MM were 57.41 years, 62.56 years, 64.73 years, and 59.28 years, respectively. Female (59%) was slightly more preponderance over the male (41%) for SGC than other malignancies. Pigmentation was associated with malignant melanoma (100%) and BCC (81%). Statistically, a significant difference was found between eyelid malignancies, including location, size, pigmentation, recurrence, and invasiveness. The recurrence rate was low lower in the patients who underwent frozen section biopsy (3%) for margin clearance than those who underwent excision biopsy (21.5%) in the follow-up time. Conjunctival map biopsy (8%) was performed as an essential tool for excluding the pagetoid spread of SGC. A new reconstruction method named triangular-shaped musculocutaneous tail flap was performed in 33 (11%) patients to reconstruct the moderate eyelid defect following local resection.

**Conclusion:**

Sebaceous gland carcinoma (SGC) was the highest occurrence found to be the highest occurrence among all eyelid malignancies in Bangladesh. SGC is more aggressive and the recurrence rate was higher than BCC and SqCC.

## 1. Introduction

Eyelid carcinoma is the most common malignant neoplasm of the eyelid region, and it may involve either the skin or the tarsus and inner layer of the eyelid [1]. In general, eyelid cancer is curable if it is managed in its earliest stages. About 50% of all malignant tumours involve the skin. About 5–10% of skin cancer occurs in the eyelid [2–5]. The most common primary eyelid malignancy is BCC which is rarely metastatic. Other carcinomas such as SqCC, SGC, malignant melanoma, and Markel cell carcinoma are associated with more spreading in nature to the surrounding structures and a more pronounced metastatic potential [3, 4, 6–8]. In the USA and Europe, the frequency of BCC is the highest (90–95%) among all eyelid malignancies, whereas SqCC and SGC make up only less than 10% [9, 10]. In Asian countries, the SGC accounts for 27% to 53% and is as common as BCC [11–20]. The prevalence of SqCC varies in different Asian countries; it is high (32%) in Pakistan and low (6%) in China [21, 22]. SGC may masquerade as recurrent chalazion or chronic blepharitis. The incidence of metastasis is high in SGC, and it may have a pagetoid/multicentric pattern [23–25]. Adverse prognostic features include involvement of the upper eyelid, a tumour size of 10 mm or more, and a duration of symptoms of over six months. Standard frozen section control biopsy and Moh's micrographic surgery are the standard biopsy techniques for negative surgical margin clearance. Conjunctival map biopsies are helpful to assess the lesions for pagetoid spread [24–26]. Management of eyelid malignancy consists of surgical excision with negative microscopic margin clearance followed by eyelid reconstruction in most instances, as well as the judicious use of adjuvant radiotherapy and topical chemotherapy in selected patients [1]. Patients with locally advanced BCC who are not amenable to definitive surgery, newer targeting therapies, or systemic treatments may be the alternative options to preserve the globe [27, 28]. However, our country has no accurate documented report on eyelid malignancies. Here, we will discuss the relative proportion of eyelid malignancies in the Bangladesh population and describe their clinical features and management outcomes. We also attempt to build awareness about the more aggressive eyelid malignancy to reduce its morbidity and mortality.

## 2. Methods

This retrospective case series study was conducted at Bangladesh Eye Hospital and Institute, Dhaka, Bangladesh, from 01 January 2020 to 31 December 2020. Institutional review board (IRB) approval was obtained. A review was carried out and data extracted from our all-recorded medical documents, and a histopathology database was made for the overall assessment of eyelid malignancies.

This study excluded patients with secondary eyelid malignancies and clinically diagnosed eyelid malignancies without histopathological proof from this research. All patients were included who were followed up at least six months after histopathological confirmation of primary eyelid malignancy from 01 January 2014 to 31 December 2019. Orbital imaging is rarely advised if the posterior margin of the tumour cannot be estimated on clinical examination and in the presence of Proptosis.

All patients were managed by surgical excision with tumor-free margins verified on histopathology, either on frozen section or excision biopsy with 2–3 mm microscopic view of normal tissue followed by eyelid reconstruction. The frozen section represents the intraoperative tumour clearance of the resected margin of the eyelid. Although no formal contraindications to the use of frozen sections exist we explained about the advantage of frozen section biopsy over excisional biopsy. Many patients preferred excisional biopsy to frozen section procedure as it is cost-effective. Conjunctival map biopsy was advised for the suspected pagetoid spread of SGC. The eyelid was meticulously reconstructed after excision or frozen section biopsy. Surgical reconstruction was performed based on the size of the eyelid defect after excision of the tumour. The following demographic and clinical variables were evaluated from our records: age, gender, and location; the pattern of malignancy; tumour size; clinical and histopathological diagnosis; eyelid reconstruction techniques; and outcomes details.

The data were compiled and analyzed statistically. Statistical analysis was used to assess the differential distribution of variables observed in this study. Computer-based statistical software SPSS (version 17) was used for the analysis. *P*value <0.05 was considered as significant and <0.001 was considered highly significant. An appropriate test of significance (chi-square test) was used for the statistical analysis.

## 3. Results

A total of one thousand seven hundred three (1703) patients with clinically and histologically confirmed eyelid tumors were reviewed during the study. Among them, benign tumors were 1371 (80.5%) and histologically confirmed malignant tumors were 332 (19.5%).

**Figure 1:**
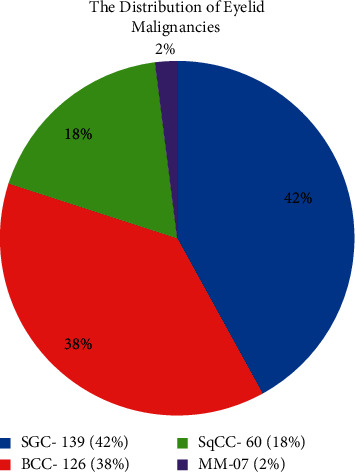
Distribution of eyelid malignant tumors.

The histopathologically proven eyelid malignancies include 139 cases (42%) of sebaceous gland carcinoma (SGC), followed by 126 cases (38%) of basal cell carcinoma (BCC), 60 (18%) cases of squamous cell carcinoma (SqCC), and 7 (2%) cases of malignant melanoma (Figure 1). SGC was the highest in frequency than others.

The mean age of all patients was 62.47 years with an age range from 34 to 96 years.

The mean age was 59.39 years, 64.56 years, 64.73 years, and 57.28 years in the cases of SGC, BCC, SqCC, and melanoma, respectively. The mean difference between SGC vs BCC, SGC vs SqCC, and BCC vs SqCC were statistically insignificant using the *t*-test.

The final analysis included 332 patients with histopathologically proven eyelid carcinoma with 178 (53.6%) male patients and 154 (46.4%) female patients. Females (86 cases, 59%) were more predominant than males in the cases of SGC. Males were more preponderant than females in the cases of BCC (60.3%), SqCC (68.3%), and MM (57%). The *P*value was highly significant (<0.001).

**Figure 2:**
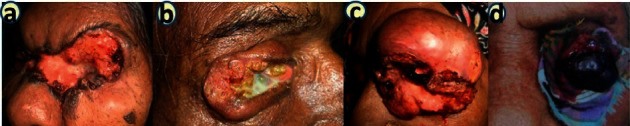
Different types of locally invasive eyelid malignancies: (a) BCC, (b) SqCC, (c) SGC, and (d) melanoma.

The size of the lesion is always an indicating factor for the occurrence of metastasis and also for recurrence. All lesions were assessed according to tumour size such as up to 0–10 mm, 11–20 mm, and more than 20 mm (Figure 2). Most of patients (245 cases, 73.8%) presented with more than 10 mm (>1 cm) eyelid lesions in size. In BCC, the number of pigmented lesions was 102 (81%). All (100%) malignant melanomas were pigmented. The upper eyelid was commonly affected in 154 (46.4%) cases, whereas the lower eyelid was involved in 124 (37.3%) cases of eyelid malignancies. The upper eyelid was commonly affected in SGC (81.3%) and the lower eyelid was commonly involved in BCC (70.6%). Local invasion into the orbit was found in 32 (9.6%) cases. Lymph node metastasis was noted in 26 (7.8%) cases and metastasis to the distant organ was assessed in 9 (2.7%) cases of eyelid malignancies. Both the lymph node and distant metastasis were found in the patients with SqCC, SGC, and malignant melanoma (Table 1).

**Figure 3:**
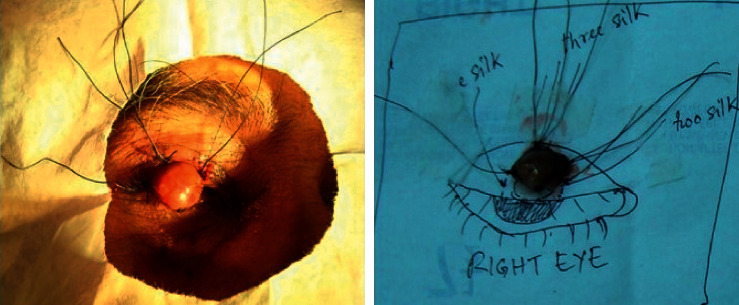
Frozen section biopsy procedure of sebaceous gland carcinoma of the right upper eyelid. Each margin of the excised tissue is fixed with a marker (silk suture) and the fresh tissue is sent to the pathologist for confirming the margin clearance.

Frozen section biopsy (Figure 3) was the preferred method and was performed in 166 (50%) cases. Excision biopsy was done in 133 (40%) cases; only incision biopsy was performed in 14 cases (4.2%). Extensive malignancies were referred to an oncologist for nonsurgical management. However, 19 (5.9%) cases of eyelid malignancy with orbital invasion were managed by surgical excision including orbital exenteration.

**Figure 4:**
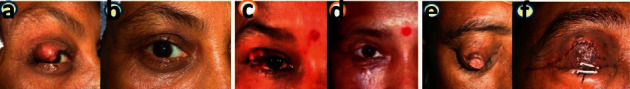
Preoperative and postoperative photographs of direct closure with canthotomy and cantholysis (a, b), semicircular flap (c, d), and preoperative with immediate images of the triangular flap (e, f).

**Figure 5:**
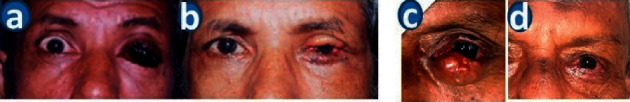
Preoperative and postoperative photographs of eyelid shearing procedures: (a, b) Cutler–Beard procedure and (c, d) Hughes procedure.

**Figure 6:**
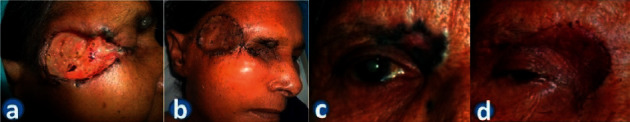
Surgical outcome (exenteration with excision biopsy followed by skin graft) of locally advanced basal cell carcinoma (a, b), and the result of excision biopsy with skin graft of morpheaform basal cell carcinoma (c, d).

Eyelid reconstruction was carried out in 299 cases (90%) of eyelid malignancies following surgical excision. The different types of eyelid reconstruction (Figure 4) were performed based on the eyelid defect following surgical excision. The most common technique of eyelid reconstruction was the lid shearing procedure (Curtler–Beard/Hughes procedure) (Figure 5) which accounted for 43.1% followed by direct closure with canthotomy and cantholysis (12%) and semicircular flap (20%). A new reconstruction procedure (tail flap) was performed in 33 (11%) cases to reconstruct the moderate eyelid defect. The lesions involving the skin (36 cases, 12%) away from the lid margin were corrected by skin graft (Figure 6), Z-plasty, or advancement flap.

Recurrences were noted in the follow-up period of 6 months of surgery. Recurrences were found in 38 (11.9%) patients, and all recurrences were noted in the cases of large lesions (>1 cm). The recurrence rate was high in the patients with SGC, at 15.6%. The recurrence rate was lowest (3%) among the patients who underwent frozen section control biopsy when compared to excision biopsy and excision biopsy with exenteration (21.5%). The chi-square value was 22.89 (*P*value <0.001). The mortality rate was 5.7% in our study.

## 4. Discussion

In a study on 4,521 eyelid lesion patients, benign tumors accounted for 95% and malignant tumours accounted for only 5% [3]. Eyelid malignancy accounts for 19.5% (332 cases) in this study. In the United States and Europe, BCC (85–95%) is the most common eyelid carcinoma, whereas others eyelid malignancies accounts 5–15% [2, 6, 10, 29–32]. The reported occurrence of SGC (37%–41%) is equal to that of BCC in Japan and India [11, 14, 19]. The prevalence of SGC is higher than BCC in various reported literature of South Asia [13, 15, 33–38]. In East Asia, the rate of BCC is higher (42% to 82%) among the eyelid malignancies [3, 17, 18, 39–46]. The highest occurrence of BCC is 83% in Iran [47] and 82% in Singapore [40]. These are different scenarios from other Asian countries. The highest frequency of SGC is 38% to 53% in India, Nepal, Bangladesh, Japan, China, and Thailand [13, 14, 17, 19, 20, 34–38, 40] (Table 2). In our 332 cases, the frequency of SGC (139 cases, 42%) was the highest in occurrence of all eyelid malignancies than BCC (126 cases, 38%), SqCC (60 cases, 18%), and MM (7 cases, 2%). SGC is higher and nearer to the rate of BCC in this study. The rate of eyelid malignancies with a high frequency of BCC is consistent with some studies from Korea, India, Japan, Taiwan, and Nepal [11, 37, 38, 44, 45, 48]. However, these studies have also shown a different scenario of the incidence of eyelid malignancies in Asian countries when compared to western countries [3, 4, 6, 11, 12, 37, 38, 43, 48].

In this study, the mean age was 61.4 years with a minimum age of 34 years and a maximum age of 96 years. SGC (71 cases, 51%) commonly occurs in the age group of 46 to 60 years, whereas BCC (69 cases, 54.7%) and SqCC (34 cases, 57.7%) are in the 61–75 year age group. Malignant eyelid tumors are rare in children and young adults but occur more commonly in the sixth, seventh, and eighth decades of life [12, 37, 38, 43–45, 48, 49]. The reported age range of the patients was 11 years to 94 years, and about 45% of the patients were aged between 60 and 70 years [31]. The incidence of BCC is higher in people over 60 years of age [31]. A prevalence of male gender was reported in patients with BCC [32, 50–53]. SGC occurred more frequently in females with a female to male ratio of 1.5 to 1.0 [47, 54]. Females (59%) are slightly more predominant among the patients with SGC in our study, while male predominance was found in the cases of BCC (60.3%), SqCC (68.3%), and malignant melanoma (57%). The reason for the female preponderance of SGC in our study is unknown, though it may be genetic cause or related to an increased rate of cosmetic use and beetle leaf and nut chewing in females.

This study reported that the lesion diameter is found more than 10 mm in 245 cases (73.8%) of eyelid carcinoma. Larger (>10 mm) eyelid malignancy is associated with poorer prognosis [53]. Pigmentation lesions are recorded in all cases of malignant melanoma and 102 (81%) cases with BCC. Pigmentation was rarely found in SqCC (5%) and SGC (1.4%). Pigmented lesions were found in 55% of the cases of BCC [38]. Pigmented BCC is commonly found in the Asian population and patients with darker skin color [54–57].

Neglected and untreated eyelid carcinoma may involve both sides of the eyelids. In our study, 4 cases of BCC (3.2%) and one case of SqCC (1.6%) involved both sides of the eyelids. Involvements of both eyelids were associated with bad prognosis in terms of metastasis and mortality [57]. The tumor epicenter was located in both eyelids of SGC (13%), BCC (3.6%), and SqCC (5%) in our study. This study reported that the upper eyelid was commonly affected in SGC (113 cases, 81.3%) but the lower eyelid (5 cases, 3.6%) was rarely affected. Most of the cases (89 cases, 70.6%) of BCC and malignant melanoma (6 cases, 85.7%) were commonly involved the lower eyelid, and the involvement of the upper eyelids was rarely found in BCC (9 cases, 7%). There was statistically significant predilection between the involvement of upper eyelid (51.7%) and lower eyelid (40%) of SqCC. The lesion affected the medial canthus in 19 cases (15.1%) of BCC and the lateral canthus in 37 cases (26.6%) of SGC in our study. About half to two-thirds of SGC most commonly arise from the upper eyelid [4]. 59% of SGC arose from the upper eyelid [38]. BCC is more common in the lower eyelid (50–66%), followed by medial canthus (10–30%), upper eyelid (15–16%), and lateral canthus (3–5%). There was no preference for upper or lower eyelids of SqCC [31, 57]. Medial canthus (48%) and lower eyelid (32%) was the most frequent involved site for BCC [51, 58, 59].

The diagnosis of eyelid tumor was confirmed by histopathological analysis with a correlation of clinical findings. Clinical diagnosis was correct in 96.3% of BCC, 83.3% of SGC, and 80% of SqCC in this research. The clinical diagnosis was commonly missed as SGC in 46% of SqCC [38].

Invasion to orbit is evaluated in 32 (9.6%) cases. The orbital invasion was evaluated in 14 cases (10%) of SGC, 11 cases (8.7%) of BCC, 5 cases (8.3%) of SqCC, and 2 cases (28.6%) of MM. Lymph node metastasis was found in 16 cases (11.5%) of SGC, 9 cases (15%) of SqCC and one case (14.3%) of MM. Metastasis to the distant organ was assessed in the 6 cases (4.3%) of SGC, 2 cases (3.3%) of SqCC, and one case (14.3%) of MM in this study. Invasion to orbit and surrounding structures could be assessed in advanced BCC, but metastasis rarely occurs in BCC. Lymph node metastasis was noted in 25% of SqCC, whereas it was noted only in 09% of BCC and 11.1% of SGC [11]. Local invasiveness (13%), lymph nodes (16%), and systemic metastasis (13%) were found with SGC, compared to SCC and BCC. SGC was more commonly associated with tumor recurrence, systemic metastasis, and mortality. Lymph node metastasis was also more common with malignant melanoma, Merkel cell carcinoma, and lymphoma [4, 12, 38, 57, 59].

The standard modality for biopsy technique is frozen section control excision biopsy or Moh's micrographic surgery control [4, 12, 60, 61]. In our study, frozen section biopsy was the preferred method rather than excision biopsy with 2–3 mm of microscopic healthy tissue. Incision biopsy was performed in 14 cases (4.2%) with extensive eyelid lesions and in cases of bilateral eyelid malignancies where total surgical excision was not amenable, patients were referred to oncologist for nonsurgical management like radiotherapy, chemotherapy, or palliative therapy. Frozen section control excision biopsy was done in 41% of SGC, 60.3% of BCC, 48.3% of SqCC, and 57.1% of malignant melanoma cases. Wide excision biopsies with 2–3 mm of healthy tissue were carried out in 46.8% of SGC, 32.5% of BCC, 41.7% of SqCC, and 28.6% melanoma of the eyelid. We performed a map biopsy in the conjunctiva to exclude the pagetoid spread of SGC (8%). Every intraepithelial spread with SGC should be evaluated by conjunctival map biopsy [9]. Radical exenteration was carried out in 19 cases, among which 58% consisted of SGC. Eyelid reconstruction is performed depending on eyelid defects following surgical excision. Small defects (<33%) were managed by direct closure with canthotomy and cantholysis in 12% of eyelid malignancies, moderate defects (33–45%) were managed by semicircular flap (60 cases, 20%) and a new technique named “tail flap” (33 cases, 11%), and large defects were corrected by lid sharing procedures (129 cases, 43.1%). The lid sharing procedures include the Curtler–Beard procedure for upper eyelid reconstruction and the Hughes procedure for lower lid reconstruction. For lower eyelid reconstruction, it is important to repair the anterior lamella and the posterior lamella separately to achieve an excellent aesthetic and functional outcome [61].

The mortality was 5.12% (17 cases) and highest in SGC (13 cases, 9.35%). The patients with systemic metastasis of eyelid malignancies died in the follow-up period. The larger lesions (>2 mm in diameter) are associated with local invasion to surrounding structures, lymph node metastasis, or systemic metastasis. So far, the patients who underwent surgical excision with histological clearance of tumours have had an excellent survival rate. Mortality was high in SGC compared to other malignant tumors [4, 25, 26, 38, 62–64]. Recurrence was noted in the 3% of cases that underwent frozen section biopsy compared with 23% of wide excision biopsy and 10.5% of the exenteration cases. Our study report is correlated with the maximum reports from Asian countries, but contrary to the American and European reports. The frequency of SGC is nearer to BCC and SGC is much higher in the Asian region than in non-Asian regions.

## 5. Conclusion

Sebaceous gland carcinoma has the highest occurrence rate in Bangladesh. The gold standard for treatment modality is surgical excision with microscopic margin clearance under frozen section control. Metastasis and recurrences are usually found in cases of SGC rather than basal cell carcinoma and squamous cell carcinoma. Melanoma is rarely occurred eyelid malignancy. This finding differs from the Western reports and is similar to those from Asian regions.

## Figures and Tables

**Table 1:** Distribution of the clinical analysis and surgical outcome of the study subjects.

Variables	SGC	BCC	SqCC	MM	Results
	No. (%)	No. (%)	No. (%)	No. (%)	
Size of the lesion					
≤1 cm	35 (25.2)	37 (29.4)	13 (21.7)	02 (28.6)	*P* value = >0.1^ns^
1–2 cm	79 (56.8)	77 (61.1)	37 (61.7)	04 (57.1)	
>2 cm	25 (18.0)	12 (09.5)	10 (16.6)	01 (14.3)	
**Pigmented lesion**	**02 (01.4)**	**102 (81.0)**	**03 (5.0)**	**07 (100)**	**Total** **=** **124**
**Bilateral**	**00**	**04 (03.2)**	**01 (1.6)**	**00**	**Total** **=** **05**

Eyelid involvement					
Upper lid	113 (81.3)	09 (7.0)	31 (51.7)	01 (14.3)	154
Lower lid	05 (03.6)	89 (70.6)	24 (40.0)	06 (85.7)	124
Both lids	18 (13.0)	04 (3.1)	03 (5.0)	00	25
Medial canthus	02 (1.4)	19 (15.1)	02 (3.3)	00	23
Lateral canthus	37 (26.6)	01 (0.8)	01 (0.8)	00	39
Periocular skin	05 (3.6)	22 (17.4)	02 (1.6)	00	29

Invasiveness					
Orbit	14 (10.0)	11 (08.7)	05 (8.3)	02 (28.6)	*P* value = >0.1^ns^
Lymph node	16 (11.5)	00	09 (15.0)	01 (14.3)	
Metastasis	06 (4.3)	00	02 (3.3)	01 (14.3)	

Surgical strategies					
Incision biopsy	06 (04.3)	05 (04.0)	03 (05.0)	00 (00)	*P* value = 0.1^ns^
Excision biopsy	65 (46.8)	41 (32.5)	25 (41.7)	02 (28.6)	
Frozen section	57 (41.0)	76 (60.3)	29 (48.3)	04 (57.1)	
Exenteration	11 (07.9)	04 (03.2)	03 (05.0)	01 (14.3)	
**Recurrences**	21 (15.8)	07 (5.7)	09 (15.7)	01 (14.3)	Total = 38, (12%)
**Deaths**	13 (09.3)	01 (0.8)	01 (01.7)	02 (28.5)	Total = 17, (5.7%)

**Table 2:** Review of the Literatures since 1991 on the ratio of eyelid malignancies in Asian countries.

Author (s), year	Country	BCC (%)	SGC (%)	SqCC (%)	Others (%)	Total no.	Most common
Wang et al. 2003 [48]	Taiwan	62	24	09	05	127	BCC
Chang et al. 2003 [39]	Taiwan	78	17	06	00	18	BCC
Obata et al. 2005 [19]	Japan	38	38	00	25	24	BCC, SGC
Pornpanich & Chindasub 2005 [20]	Thailand	41	38	06	15	32	BCC
Takamura and Yamashita 2005 [18]	Japan	40	29	11	21	38	BCC
Lin et al. 2006 [43]	Taiwan	65	08	13	14	1121	BCC
Jahagirdar et al. 2007 [11]	India	44	37	15	04	27	BCC
Xu et al. 2008 [17]	China	41	39	05	15	363	BCC
Kumar 2010 [35]	Nepal	24	41	27	08	37	SGC
Mak et al. 2011 [46]	China	75	11	06	08	36	BCC
Kale et al. 2012 [21]	India	48	31	14	07	85	BCC
Lim and Amrith 2012 [40]	Singapore	82	11	04	05	333	BCC
Wang et al. 2013 [16]	China,& Korea	48	29	09	15	75	BCC
Hussain et al. 2013 [22]	Pakistan	59	07	32	03	222	BCC
Bagheri et al. 2013 [47]	Iran	83	06	08	03	100	BCC
Ho et al. 2013 [42]	China	43	07	18	32	28	BCC
Ramya et al. 2014 [37]	India	27	41	22	10	24	SGC
Krishnamurthy et al. 2014 [15]	India	26	32	21	21	19	SGC
Kadir et al. 2014 [34]	Bangladesh	40	41	17	02	164	SGC
Park et al. 2014 [41]	Korea	56	15	23	06	73	BCC
Rathod et al. 2015 [14]	India	41	41	10	08	39	BCC, SGC
Huang et al. 2015 [3]	Taiwan	58	21	10	7.5	227	BCC
Mohan and Letha 2017 [36]	India	15	24	22	39	41	SGC
Jangir et al. 2017 [13]	India	29	47	24	0	17	SGC
Kaliki et al. 2019 [38]	India	18	53	24	04	536	SGC
Kadir SMU et. al. 2020 (Present study)	Bangladesh	38	42	18	02	332	SGC

## Data Availability

The data are available from the corresponding author upon reasonable request.

## Abbreviations

SGC: Sebaceous gland carcinoma
BCC: Basal cell carcinoma
SqCC: Squamous cell carcinoma
MM: Malignant melanoma.
